# Testing the discrepancy between actual and ideal body image with the Implicit Relational Assessment Procedure (IRAP)

**DOI:** 10.1186/s40337-021-00434-4

**Published:** 2021-07-08

**Authors:** Mónica Hernández-López, Lourdes Quiñones-Jiménez, Alberto L. Blanco-Romero, Miguel Rodríguez-Valverde

**Affiliations:** Department of Psychology, University of Jaén, Campus las Lagunillas s/n, 23071 Jaén, Spain

**Keywords:** Figural drawings, Body image, Body dissatisfaction, Implicit attitudes, Implicit relational assessment procedure, IRAP, Gender differences

## Abstract

**Background:**

The discrepancy between actual and ideal body image is considered an index of body dissatisfaction and a risk factor for eating disorders. While discrepancy has been traditionally tested with figural drawing rating scales, in recent times the use of implicit measures has been explored.

**Methods:**

This study employs the Implicit Relational Assessment Procedure (IRAP) to examine actual-ideal body-size discrepancy in a sample of 130 Spanish college students, as well as its utility to predict symptoms of eating disorders and other body-image relevant measures. Participants completed the Contour Drawing Rating Scale (CDRS). The three smallest and the three largest contour drawings of the CDRS were used as target stimuli in two different IRAP tasks: one in combination with the sample phrases “I am” and “I am not” (that assessed implicit actual body image), another in combination with the phrases “I want to be” and “I don’t want to be” (that assessed implicit ideal body image). After completing both IRAP tasks, participants completed explicit measures of body-image psychological inflexibility, body dissatisfaction, and symptoms of eating disorders.

**Results:**

Results showed a small implicit bias towards thinness. Participants were faster in affirming than denying that they are thin and that they desire to be thin. They were also faster in affirming than denying that they are fat and that they want to be fat, but to a smaller extent than with thinness. Specifically, the implicit desire to be (or not be) fat emerged as an independent predictor of eating disorder symptoms, psychological inflexibility, and body dissatisfaction that significantly increased the predictive power of CDRS scores.

**Conclusions:**

These findings underscore the need for further research on specific body image implicit beliefs towards fatness, both in subclinical and clinical populations, in order to examine whether willingness to accept the idea that one can have a larger body size can be a suitable target for prevention and intervention in eating disorders.

## Plain English summary

Body dissatisfaction can be thought of as the discrepancy between actual and desired body size. This is typically measured with rating scales depicting a series of body silhouettes of increasing size, from extreme thinness to fatness. Respondents mark the points that match their current body size and the one they would ideally like to be (the more distance between both, the more body dissatisfaction). Like all self-reports, these scales are liable to self-presentation biases. Experimental procedures that require respondents to answer under time pressure (implicit measures) tap into more automatic reactions that are less amenable to deliberate manipulation. This study used one such procedure (the IRAP) to examine how rapidly participants identified themselves (or not) with pictures of underweight or overweight, and how rapidly they expressed a desire to be (or not) like such pictures. Participants did not show a clear discrepancy between their actual and their ideal body size with the IRAP. However, automatic reactions indicative of acceptance/rejection of the idea of becoming fatter, predicted the degree of body image distress as measured with different instruments. Unwillingness to accept this idea was associated with more severe symptoms of disordered eating.

## Background

The discrepancy between actual body image (perceived current body size) and ideal body image (desired body size) is frequently employed as a measure of body dissatisfaction [1, 2]. Most frequently, this discrepancy consists of desiring a thinner body. This is particularly the case for women [3]. A vast literature provides evidence that this desire for a thinner body is generalized, especially in Western societies [4, 5].

According to self-discrepancy theory, perceiving one’s body to be larger than desired might lead to dissatisfaction and depression due to unfulfilled hopes and wishes. These emotional reactions would also motivate behavior (including problematic eating behavior) aimed at reducing this body image discrepancy [3]. The emotional and behavioral impact of actual-ideal body size discrepancy, however, can be different across individuals depending on other variables. For instance, the extent to which physical attributes and appearance are relevant to self-identity, with individuals more concerned with physical appearance experiencing more aversive thoughts and emotions [6]. In addition, how flexibly individuals respond to their own cognitive and affective states related to body shape (i.e. body-image psychological flexibility [7, 8]) seems to play a relevant role too. Being open to experiencing unpleasant thoughts and emotions about one’s body size in a detached way, without attempting to control them, limits how aversively these thoughts and emotions are experienced and the degree of control they exert over eating behavior [9, 10]. Therefore, while body dissatisfaction is clearly associated with unhealthy eating habits and is a risk factor for the emergence and maintenance of eating disorders [11], it appears that psychological (in)flexibility regarding one’s body size can mediate how body dissatisfaction leads to problematic eating behavior [12].

Figural drawing rating scales are one of the most common ways of evaluating actual-ideal body size discrepancy [1, 2]. These instruments typically consist of silhouette or schematic drawings of human bodies varying in body shape or size, from extreme thinness to obesity. Respondents are asked to select the figure drawing that matches their current body size (actual body size), and then to select the drawing that best represents the body size they would like to be (ideal body size). The difference between both responses is calculated as a discrepancy score. These scales are very popular, since they are easy to apply and correlate strongly with self-report measures of disordered eating [13]. However, they are not without criticism. It has been pointed out that the presentation of all the figure drawings on a single sheet in ascending size might lead participants to respond to a reduced subset of all available figures, thus reducing variability and spuriously increasing test-retest reliability [14, 15]. In addition, the presentation order of the scales (actual followed by ideal) as well as the instructions provided, might induce participants to select a thinner ideal figure, assuming a demand characteristic based on societal standards [16]. This latter criticism, that respondents might be biased by demand characteristics, is applicable to other explicit self-report measures, like questionnaires. Despite their usefulness and convenience in obtaining information from large samples, explicit self-report measures are liable to self-presentation biases. Respondents might craft their answers in order to present themselves in a socially desirable manner, in accordance with the social norm, or just try to respond according to what they believe the researcher is expecting from them [17]. In addition, it is unclear to which extent individuals can accurately introspect and be aware of their own beliefs and attitudes [18, 19]. These limitations have been addressed by employing the so-called implicit measures (experimental procedures based on response latency and response accuracy under time-pressure conditions), which are supposed to assess automatic, immediate attitudes and beliefs [20]. They do not require the participant to deliberately evaluate their preference and consciously produce a value judgment, but their attitudes are inferred from performance in the experimental task. A growing literature is providing evidence about the utility of implicit attitudes for the study of body image and eating behavior [21].

One such implicit measure that seems particularly well suited for the assessment of actual-ideal discrepancy is the Implicit Relational Assessment Procedure (IRAP [22]). The IRAP is conceptually based on a functional-contextual approach to the study of language and cognition, Relational Frame Theory (RFT [23, 24]). Unlike other implicit measures that are based on associative conceptual models, the IRAP is a procedure designed for the assessment of verbal relations or propositions in flight. It is assumed that participants have a long pre-experimental learning history of relating the relevant stimuli in the task (e.g., “I” and “thinness”) in specific ways. The IRAP assesses how participants respond to different types of relations amongst these stimuli, with the assumption that participants will be faster in responding to the types of relations that are consistent with their learning history (i.e., the verbal responses that have been more strongly reinforced, or in cognitive terminology, the individual’s personal beliefs). For instance, consider an overweight teenager who has been exposed throughout her life to criticism by relatives and peers, who has been repeatedly told that she should lose weight so that she would look better and be healthier, and who has been massively exposed through the media to slim models and influencers representing beauty and success. It is likely that this person will be faster in affirming than denying that she wants to be thin, and that she will be faster in denying than affirming that she wants to be fat (i.e. showing an implicit desire for thinness). Crucially, as pointed out by Heider et al. [25], the beliefs involved in actual-ideal body size discrepancy can be best thought of as verbal relations (e.g. “I am fat”, or “I don’t want to be fat”), rather than merely as associations (e.g. “I-fat”).

The IRAP has already been successfully used for the assessment of actual-ideal discrepancy related to body image. Heider et al. [25] examined actual and ideal implicit body image in two groups of Belgian female college students with extreme (high vs. low) self-reported scores in body dissatisfaction. Participants underwent two different IRAP tasks devised to capture actual and ideal body image. In the first one, participants were presented with combinations of the phrases “I am” and “I am not” with words referring to the concepts of thinness and overweight (e.g., slim, chubby, etc.). Half of the trials in each block presented combinations that accorded to the verbal relations I am thin/I am not fat, and the other half accorded to the verbal relations I am fat/I am not thin. On half of the trial-blocks, participants were asked to respond, as fast as possible, as if they were thin. They were thus expected to select “True” for stimulus combinations such as “I am slim” and “I am not chubby”, and “False” for combinations such as “I am chubby” and “I am not slim”. On the other half, they were asked to respond as if they were fat (i.e., by selecting “True” for stimulus combinations such as “I am chubby” and “I am not slim”, and “False” for combinations such as “I am slim” and “I am not chubby”). The second IRAP task (ideal body image) was very similar, but the phrases “I want to be” and “I don’t want to be” were used instead, with the same target words referred to thinness and fatness. Participants were asked to respond as if they wanted to be thin on half of the trial-blocks, and as if they wanted to be fat on the other half. For each IRAP task, a score was calculated based on the difference between average latencies to both types of trial blocks. Heider and colleagues found that participants with low levels of body dissatisfaction endorsed the implicit belief that they are thin (and not fat) more strongly than participants high in body dissatisfaction. Conversely, they found the latter to show a stronger implicit desire to be thin (and not fat) than the former. However, when testing whether self-reported body dissatisfaction could be predicted on the basis of actual-ideal body image discrepancy (in a hierarchical linear-regression model), they found that IRAP scores did not add to the predictive power of an explicit measure of discrepancy (the contour drawing rating scale, CDRS [26]).

One way to further explore this interesting line of inquiry is by examining whether a more nuanced analysis of actual and ideal body image can be undertaken based on the findings of IRAP research on implicit attitudes to others’ body size [27–31]. These studies conducted more fine-grained analyses based on the calculation of specific scores for each trial type or for each category (e.g., Thin vs. Fat, with separate scores for trials presenting thin models, and trials presenting overweight models), in addition to an overall, relative preference score like the one calculated by Heider et al. [25]. It is noteworthy that none of these studies found implicit anti-fat attitudes. Some of them, with samples comprising both female and male participants [29–31] found an implicit weight bias (an overall evaluative preference for pictures of thin models over pictures of overweight models) that was entirely attributable to pro-thin attitudes and not to anti-fat ones (i.e., scores based on trials presenting overweight models were indicative of a neutral attitude). Other studies, with female-only samples [27, 28] did not find any implicit bias. Participants in Maroto-Expósito et al. [28] showed positive attitudes of similar magnitude both to pictures of underweight and pictures of overweight models. This pattern of results was replicated in a later study from the same research group [27], but only for participants with very low levels of body dissatisfaction. Participants with high levels of body dissatisfaction showed a preference for underweight model pictures similar to that found in the aforementioned studies by Roddy and colleagues [30, 31] (i.e. a positive attitude to thinness and a neutral attitude to fatness). In sum, previous IRAP research suggests that, at least regarding others’ body size, while positive implicit attitudes to thinness seem widespread, attitudes to fatness vary from neutral to positive, depending on individual differences (e.g. gender, previous levels of self-reported body dissatisfaction). Specifically, the evidence suggests that it is implicit attitudes to fatness that play a significant role in the prediction of different clinically relevant body-image outcomes (body-image psychological inflexibility, body dissatisfaction, and eating disorder symptoms) (see [27]). Accordingly, we believe it is relevant to further examine the role of implicit attitudes to fatness in actual-ideal body size discrepancy (e.g., to which extent an individual is open to responding as if they want to be fat). In our view, the verbal relations involved in these implicit attitudes would characterize (in)flexibility towards one’s body image. It seems that psychological flexibility regarding body image would entail being open to (and not automatically reject) the idea of being fat.

The present study aims to examine implicit actual-ideal body image through IRAP tasks that more closely resemble traditional measures of body-image discrepancy. To that end, two different IRAP tasks were used, one to capture actual body image (i.e. current body size) and one to capture ideal body image (i.e. desired body size), using figural drawings of underweight and overweight/obesity as target stimuli. A sample of female and male college students completed a figural drawing rating scale, the two IRAP tasks, and self-report measures of body-image psychological inflexibility, body dissatisfaction, and eating disorder symptoms. The study examines IRAP scores specific to a thin body image and to a fat body image, and explores their relationship with the other body-image measures. Secondarily, it explores gender differences in implicit and explicit actual-ideal body image discrepancy. Based on previous IRAP research on implicit attitudes to others’ body size, we expect to find a clear endorsement of the idea of being thin (actual) and of the desire to be thin (ideal). Likewise, we do not expect participants to reject the idea of being fat, or the desire to be fat. We also expect that responding to verbal relations specific to fatness will be more strongly associated with and will be predictive of self-report measures of body-image, particularly body-image psychological inflexibility. Regarding gender differences, we expect females to show larger actual-ideal body size discrepancy as measured with the figural drawing rating scale, but we refrain from making predictions about gender differences on performance on the IRAP tasks, since no previous study has explored actual-ideal body image with the IRAP in male samples.

## Methods

### Participants

One hundred and thirty students at University of Jaén (a mid-sized public university in the Andalucía region, Spain) took part in the study in exchange for course-credit. Of those 130, 107 (82.3%) completed all measures and produced valid data in both IRAP tasks, thus constituting the final sample of the study (59% female). Participants’ ages ranged between 18 and 29 years old (*M* = 19.95; *SD* = 2.31), and their average body mass index (BMI) was in the normal weight range (*M* = 22.96; *SD* = 4.08). None of them self-reported diagnoses of eating disorders or other severe psychopathologies, nor had any experience with implicit measures.

The final sample size allowed for a minimum statistical power (1-ß) of .80 for medium effect sizes for all statistical tests performed in the data analyses, as calculated with G*Power software (version 3.1).

### Self-report measures

- Demographics and anthropometric measures. A paper-and-pencil form with open-ended questions about age, education, current or past diagnoses of eating disorders or other severe psychological disorders, weight and height. Body-mass index (weight/height^2^) (kg/m^2^) was calculated for each participant based on self-reported weight and height.

- Contour Drawing Rating Scale (CDRS [26]). The CDRS consists of two sets of 9 front-view schematic figure drawings, one with female figures (for use with female participants) and another with male figures (for use with male participants). The nine drawings are ordered so that each figure, maintaining the same height, gradually increases in body size from 1 (the underweight extreme) to 9 (the overweight/obesity extreme). Drawings 1–3 represent different degrees of underweight, drawings 4–6 are representative of different degrees of the normal weight range, and the last three (7–9) represent increasing degrees of overweight and obesity. Responding consists of marking on a horizontal 200 mm line below the nine drawings the point that best depicts the respondent’s body size. Each participant completed the CDRS twice, once for actual body size (the point that matches the body size they think they are), and once for ideal body size (the point that best depicts the body size they would like to be). A discrepancy index between actual and ideal body size is calculated by subtracting the ideal body size mark from the actual body size mark, with larger discrepancies indicative of more body dissatisfaction. The CDRS has good test-retest reliability (*r* = 0.78 [24]) and construct validity, with moderate to strong associations with measures of body dissatisfaction and BMI.

- Body Image-Acceptance and Action Questionnaire (BI-AAQ [7]). The BI-AAQ is a 12-item self-report scale that assesses psychological inflexibility regarding one’s body image, that is, unwillingness to experience unwanted thoughts and emotions about one’s body shape and/or weight. Items are rated on a 7-point Likert scale (1: never true, 7: always true). We translated the original questionnaire into Spanish and the measure revealed good internal consistency (Cronbach’s α = 0.95 [27]).

- Body Shape Questionnaire (BSQ [32]). The BSQ comprises 34-items that assess dissatisfaction and concern with one’s own body-shape (including fear of gaining weight, desire to lose weight, and low self-steem based on body shape). The items are answered on a 6-point Likert scale (1 never, 6 always). The Spanish version used in this study [33] has good psychometric properties, with high internal consistency (α = .97 [25]), and good concurrent validity with other eating disorder measures [34].

- The Eating Attitudes Test (EAT-40 [35, 36]). The EAT-40 is a 40-item questionnaire that assesses symptoms of eating disorders (anorexia and bulimia nervosa). Each statement is answered on a 6-point Likert scale, with the three highest options (always, usually, often) respectively scoring 3, 2, or 1 points, and the remaining three options scoring 0 points. The Spanish version [37] used in this study has shown good internal consistency (Cronbach’s α = .89 [27]).

### IRAP tasks

The IRAP is a reaction-time based computerized tool for the direct assessment of brief, immediate relational responding (we used the IRAP version 2012 programmed in Microsoft Visual Basic 6.0 on a Windows-based computer with a 19-in. LCD screen, 1024 × 768 pixels). Two different IRAP tasks were used in the present study. The first one (IRAP_ACTUAL_) aimed at capturing current body size and the second one (IRAP_IDEAL_) aimed at capturing ideal or desired body size.

For the IRAP_ACTUAL_ the phrases “*I am”* and “*I am not”* served as sample stimuli. Six CDRS drawings were used as target stimuli: the three underweight (drawings 1–3) and the three overweight/obese (drawings 7–9). Female figures were used for female participants, and male figures for male participants. The size of each drawing was 187 × 576 pixels (150 ppi) (black lines over a white background). Each trial consisted of a combination of one sample and one target. The sample stimulus was presented at the top center of the screen, a contour drawing was presented under the sample, in the middle of the screen, and two response options were visually prompted by phrases placed at the bottom left (the phrase “Please press D for True”) and the bottom right (“Please press K for False”) corners of the screen. Participants were required to indicate, as fast as possible, the relation between the sample and the drawing by pressing the pertinent keyboard key. If they took longer than 2000 ms to respond, the words “Too slow!” appeared and remained on screen until the participant pressed either “D” or “K” on the computer keyboard. A correct response started a 400 ms inter-trial interval where the screen went blank, followed by the presentation of another trial. An incorrect response produced a red “X” that remained in the middle of the screen until the participant gave the correct response for that trial.

In each block of trials, each sample was combined twice with each one of the six target pictures. This amounted to a total of 24 trials per block, two trials with each sample-target combination (six per trial-type: I am-thin; I am-fat; I am not-thin; I am not-fat). Blocks were always presented in pairs. In the first block of each pair, participants were instructed to respond as if they are very thin (consistent block), and in the second block they were instructed to respond as if they are fat (inconsistent block). The configuration of trials was identical across consistent and inconsistent blocks. Participants just had to respond differently across blocks. In consistent blocks, they had to respond in accordance with the rule “I am thin” (i.e. I am/underweight drawing – True; I am not/underweight drawing – False; I am/overweight drawing – False; I am not/overweight drawing– True). In inconsistent blocks, they had to respond in accordance with the rule “I am fat” (i.e. I am/underweight drawing – False; I am not/underweight drawing – True; I am/overweight drawing – True; I am not/overweight drawing– False). Feedback about accuracy (percentage of correct responses) and speed (median response latency) was presented on screen after each block. Also, each block was preceded by the presentation on screen of a written reminder about the rule for correct responding in that block (i.e., “Please respond as if you are very thin” for consistent blocks, and “Please respond as if you are fat” for inconsistent blocks).

The IRAP task consisted of a practice phase and a test phase. The practice phase comprised a minimum of one and a maximum of six pairs of practice blocks. When participants achieved an accuracy of more than 79% and a median response latency of less than 2000 ms in both blocks of the same pair, they passed to the test phase. Otherwise another pair of practice blocks was presented until participants reached the threshold criteria. If participants failed to meet criteria within six pairs of blocks, they finished their participation and their data were discarded. The test phase comprised three pairs of blocks of trials. Trials were identical to those in the practice phase.

The IRAP_IDEAL_ was identical to the IRAP_ACTUAL_ with the exception that the samples were the phrases “I want to be” and “I don’t want to be” (instead of “I am” and “I am not”), and participants had to respond according to the rules “I want to be thin” (in consistent blocks) and “I want to be fat” (in inconsistent blocks).

### Procedure

All procedures were approved by the University Ethics Review Board. Participants were recruited through in-class announcements and received course credit as compensation for participation. Participants signed a statement of informed consent upon explanation of the study features. They underwent the procedure individually in a sound-attenuated experimental cubicle. First, participants completed the CDRS_ACTUAL_ and then the CDRS_IDEAL_, as explained in the Measures section. Then they completed both IRAP tasks (first IRAP_ACTUAL_ followed by IRAP_IDEAL_). The IRAP program began with the on-screen presentation of task instructions. Comprehension was assessed by requesting participants to briefly explain the task. Upon completion of both IRAP tasks, participants completed the remaining measures (in paper-pencil format) in the following order: BI-AAQ, BSQ, EAT and the demographic and anthropometric measures. The complete procedure took around 45–60 min.

### Data preparation and data analysis plan

Participants who met the accuracy and latency criteria during the practice phase and maintained the latency criterion and a minimum 75% correct responses in at least two pairs of blocks of the test phase in both IRAP tasks were included in data analyses. Response latency (time elapsed, in ms, between the onset of visual stimulus presentation on screen and the emission of a correct response by the participant) is the primary datum of the IRAP. Following standard practice, the *D*-IRAP score, an adaptation of the *D*_*1*_-algorithm [38], a variant of Cohen’s *d* (see [39] for a detailed description), was used to quantify the magnitude of differences in response latencies between both IRAP conditions (consistent vs. inconsistent: for the IRAP_ACTUAL_ I am-thin/I am not fat vs. I am fat/I am not thin; for the IRAP_IDEAL_ I want to be thin/I don’t want to be fat vs. I want to be fat/I don’t want to be thin). The steps for this calculation (for each IRAP task) were as follows: (1) only latency data from the valid pairs of test blocks were used; (2) latencies over 10,000 ms were removed from the dataset (0% latencies removed); (3) all data from any single participant were removed if they had more than 10% of test-block trials with latencies less than 300 ms (0% participants lost to this step); (4) one standard deviation was computed for each of the four trial types and valid test blocks (a total of 12 SDs in case all test blocks were valid, and only 8 SDs in case only two pairs of test blocks were valid): four for all latencies in test blocks 1 and 2, four for test blocks 3 and 4, and four for test blocks 5 and 6; (5) for each of the valid test blocks, four mean latencies were calculated, one per trial type; (6) for each pair of test blocks and trial type, the mean latency of the consistent block was subtracted from the mean latency of the corresponding inconsistent block (one per trial type and valid pair of blocks); (7) each difference score was divided by its corresponding standard deviation from step 4, (8) the three scores for each trial type (one per test-block pair) were averaged to produce a single *D*-IRAP score per trial type; (9) the *D*-IRAP scores for the two trial types presenting underweight schematic figure drawings were averaged, and the same was done for the scores of trial types presenting overweight/obesity figure drawings, which produced a single *D*-IRAP score to each body size category and IRAP task (Actual-*D*_*thin*_ and Actual-*D*_*fat*_ for the IRAP_ACTUAL_*;* Ideal-*D*_*thin*_ and Ideal- *D*_*fat*_ for the IRAP_IDEAL_); (10) an overall relative *D*-IRAP score was calculated for each IRAP task by averaging the corresponding *D*_*thin*_ and *D*_*fat*_ scores for each task (rendering: Actual-*D*_*general*_ for the IRAP_ACTUAL_ and Ideal-*D*_*general*_ for the IRAP_IDEAL_). Scores were calculated so that positive scores indicate endorsement of a thin body image belief (either actual or ideal) and non-endorsement of a fat body image belief, and vice versa for negative scores.

A number of statistical analyses were conducted in line with previous IRAP research on body image (e.g. [27]). In order to determine whether participants endorsed thin and/or fat body-image beliefs in each IRAP task (actual or ideal), we conducted one-sample *t* tests for all mean *D*-IRAP scores against a population reference value of zero. For each IRAP task, a Bonferroni-adjusted level of α was set at .0167 (three tests per IRAP task, one for each score). Likewise, a one-sample *t* test (against zero) was conducted for the explicit mean CDRS discrepancy score. Additionally, in order to determine whether participants responded differently across IRAP tasks, a 2 (IRAP task: Actual vs. Ideal) × 2 (*D*-IRAP: Thin vs. Fat) repeated measures (Greenhouse-Geisser corrected) ANOVA was conducted.

Potential gender differences in responding to the IRAP tasks were explored by means of 2 (Gender) × 2 (*D*-IRAP: Thin vs. Fat) repeated measures (Greenhouse-Geisser corrected) ANOVAs on the data from each IRAP task, with potential post-hoc comparisons to be assessed by means of Bonferroni-adjusted (α < .025) independent sample *t* tests comparing each specific *D*-IRAP score (Thin vs. Fat) across gender. Gender differences in explicit actual-ideal discrepancy were tested through an independent-samples *t* test for CDRS scores.

Finally, associations amongst implicit and explicit measures were examined through Pearson product-to-moment correlations amongst specific *D*-IRAP scores, CDRS discrepancy score, self-report measures (BI-AAQ, BSQ, EAT) and BMI. In order to explore whether implicit verbal relations specific to fatness could be used to make clinically relevant predictions regarding body image above and beyond explicit actual-ideal body image discrepancy (as measured with the CDRS), we conducted hierarchical regression analyses with body image psychological inflexibility (BI-AAQ scores), body dissatisfaction (BSQ scores), and eating disorder symptoms (EAT-40 scores) as the criterion variables. For each analysis, we compared a model in which the criterion variable was predicted by CDRS discrepancy scores with a model that also included Ideal-*D*_*fat*_ scores as a predictor.

## Results

**Fig. 1:**
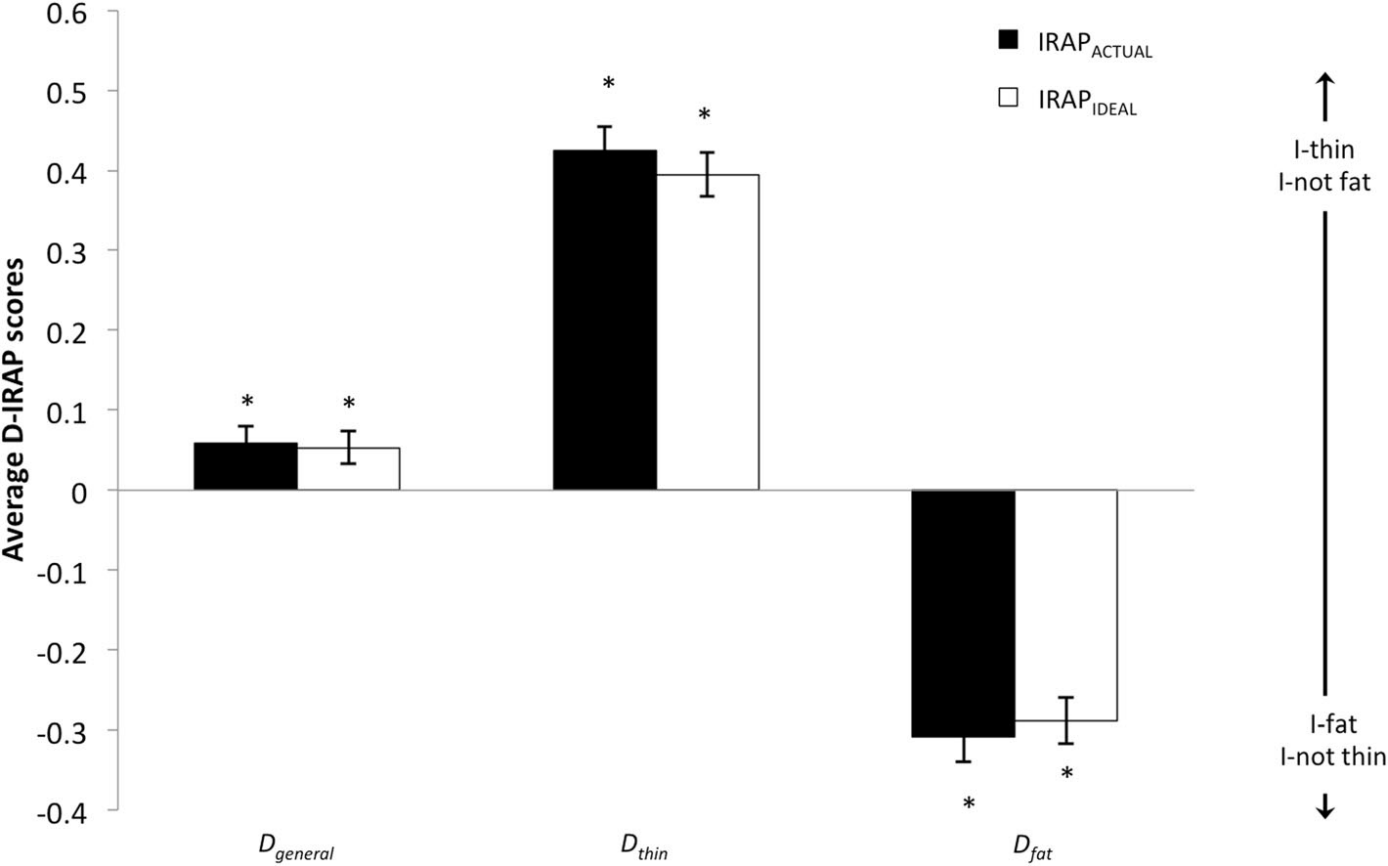
Mean (plus/minus s.e.m.) *D*_general_ (all trial types) and specific *D*-IRAP scores (*D*_*thin*_ for trials presenting schematic figures of underweight; *D*_*fat*_ for trials presenting schematic figures of overweight/obesity) for both IRAP tasks (IRAP_ACTUAL_ and IRAP_IDEAL_). Positive scores are indicative of identification with (IRAP_ACTUAL_) or desire for (IRAP_IDEAL_) thinness and not fatness, and negative scores are indicative of identification with (IRAP_ACTUAL_) or desire for (IRAP_IDEAL_) fatness and not thinness. Asterisks indicate that the score is significantly different from zero (*p* < .0167)

Figure 1 presents the averages for each *D*-IRAP score per IRAP task (IRAP_ACTUAL_ and IRAP_IDEAL_). In order to determine if *D*-IRAP scores were significantly different from zero, one-sample *t*-tests were conducted. For the IRAP_ACTUAL_, the Actual-*D*_*general*_ score (averaging all trial-types) was .059 (*SD* = .219), and it was significantly different from zero (*t*_(106)_ = 2.776, *p* = .007, *d* = .269), which is indicative of a small-sized stronger current self-identification with underweight figure drawings than with overweight/obese figure drawings. Both the Actual-*D*_*thin*_ score (*M* = .425; *SD* = .309; *t*_(106)_ = 14.207, *p* < .0005, *d* = 1.375), that only computes trial types including underweight figure drawings, and the Actual-*D*_*fat*_ score (*M* = −.308; *SD* = .330; *t*_(106)_ = − 9.652, *p* < .0005, *d* = 0.933), that only computes trial types including underweight/obesity figure drawings, were significantly different from zero too. According to this, participants were faster in affirming than denying that they are thin (when presented with trials containing any of the three underweight CDRS figures), and also in affirming than denying that they are fat (when presented with trials containing any of the three overweight/obesity CDRS figures), with a slightly stronger endorsement of the former over the latter. Similarly, for the IRAP_IDEAL_ the Ideal-*D*_*general*_ score was .053 (*SD* = .207; *t*_(106)_ = 2.670, *p* = .009, *d* = .256), which is indicative of a small-sized though significantly stronger desire to be thinner than to be fatter. Both the Ideal-*D*_*thin*_ score (*M* = .395; *SD* = .289; *t*_(106)_ = 14.155, *p* < .0005, *d* = 1.367) and the Ideal-*D*_*fat*_ score (*M* = −.289; *SD* = .294; *t*_(106)_ = − 10.135, *p* < .0005, *d* = 0.983) were as well significantly different from zero. Accordingly, participants affirmed faster than denied that they want to be thin, and also affirmed faster than denied that they want to be fat, with a slightly stronger endorsement of the former over the latter.

In order to determine whether participants responded differently to both IRAPs, a 2 (IRAP task: Actual vs. Ideal) × 2 (*D*-IRAP: Thin vs. Fat) repeated measures (Greenhouse-Geisser corrected) ANOVA was conducted. There was a significant effect for the *D*-IRAP score type (*F*_(1, 106)_ = 406.892, *p* < .0005, η^2^_p_ = .793), but neither the IRAP task (actual vs. ideal) (*F*_(1, 106)_ = .032, *p* < .858, η^2^_*p*_ < .001) nor the interaction (*F*_(1, 106)_ = 1.053, *p* < .307, η^2^_p_ = .010) were significant. Participants responded similarly in both IRAP tasks, with positive scores for trial types involving a thin body-image belief and negative scores for trials involving a fat body-image belief (both for actual and ideal body image). The lack of differential responding between the IRAP_ACTUAL_ and the IRAP_IDEAL_ contrasts with the significant positive CDRS actual-ideal discrepancy score (*M* = 18.327; *SD* = 26.691; *t*_(106)_ = 7.103, *p* < .0005, *d* = .687), which is indicative that participants explicitly desired to be thinner than they recognize themselves to be. Nevertheless, it is worth mentioning that this average CDRS discrepancy score was small in magnitude (< 10% of the 200 mm scale range) and indeed smaller than the distance between two adjacent figures in the scale.

Although the purpose of the present study was not to systematically study gender differences, we explored them by conducting 2 (Gender) × 2 (*D*-IRAP: Thin vs. Fat) repeated measures (Greenhouse-Geisser corrected) ANOVAs on the data from each IRAP task. For the IRAP_ACTUAL_, there was a significant main effect for *D*-IRAP score type (*F*_(1, 105)_ = 258.440, *p* < .0005, η^2^_p_ = .711), but neither Gender nor the interaction were significant (both *F*s < 1). For the IRAP_IDEAL_, however, in addition to the significant main effect for *D*-IRAP score type (*F*_(1, 105)_ = 320.460, *p* < .0005, η^2^_p_ = .753), there was a significant Gender × *D*-IRAP score type interaction (*F*_(1, 105)_ = 7.121, *p* = .009, η^2^_p_ = .064), though not a Gender main effect (*F*_(1, 105)_ = 1.364, *p* = .245, η^2^_p_ = .013). Independent samples *t* tests were conducted as post-hoc comparisons for gender differences in each *D*-IRAP score type. There was a significant difference for the Ideal-*D*_*fat*_ (*t*_(105)_ = 2.708, *p* = .008, *d* =. 0.538), but not for the Ideal-*D*_*thin*_ score (*t*_(105)_ = 1.009, *p* = .315, *d* = .197). While both male (*M* = −.378; *SD* = 0.264) and female (*M* = − 0.226; *SD* = 0.3) participants endorsed the implicit belief that they want to be fat, males did so more strongly. Regarding explicit actual-ideal body image discrepancy as measured with the CDRS, females (*M* = 22.098; *SD* = 26.013) showed larger discrepancy scores than males (*M* = 12.928; *SD* = 27.016), but this difference did not reach statistical significance (*t*_(105)_ = 1.766, *p* = .08, *d* = .346).

For exploratory reasons, we calculated Pearson product-to-moment correlation coefficients amongst specific *D*-IRAP scores, CDRS discrepancy score, self-report measures (BI-AAQ, BSQ, EAT) and BMI (see Table 1).

**Table 1:** Pearson product-to-moment correlation coefficients (*r*) between *D*-IRAP scores, CDRS discrepancy scores, and explicit measures (*N* = 107)

	*A-D*_*thin*_	*A-D*_*fat*_	*I-D*_*thin*_	*I-D*_*fat*_	CDRS	BI-AAQ	BSQ	EAT-40	BMI
*A-D*_*thin*_	–								
*A-D*_*fat*_	−.066	–							
*I-D*_*thin*_	.136	−.217^*^	–						
*I-D*_*fat*_	−.161	.237^*^	.008	–					
CDRS	.168	.020	−.008	−.055	–				
BI-AAQ	−.032	.046	.002	.222^*^	.408^**^	–			
BSQ	.001	.063	.015	.181^ms^	.553^**^	.817^**^	–		
EAT-40	.070	.120	.025	.207^*^	.263^**^	.614^**^	.705^**^	–	
BMI	.072	−.041	.032	−.082	.587^**^	.202^*^	.270^*^	−.036	–

*A-D*_*thin*_ Actual-D_thin_ score, *A-D*_*fat*_ Actual-D_fat_ score, *I-D*_*thin*_ Ideal-D_thin_ score, *I-D*_*fat*_ Ideal-D_fat_ score, *CDRS* Contour Drawing Rating Scale discrepancy score, *BI-AAQ* Body Image Acceptance and Action Questionnaire, *BSQ* Body Shape Questionnaire, *EAT* Eating Attitudes Test, *BMI* Body Mass Index

* p < .05; ** p < .01; ^*ms*^*: marginally significant, p = .062*

The discrepancy score of the CDRS was positively correlated with all self-report measures of body image distress, and with BMI. The larger the difference between perceived current body size and desired body size (i.e., the thinner participants want to be compared to what they perceive themselves to be), the more body image psychological inflexibility, body dissatisfaction (as measured with the BSQ) and symptoms of eating disorders participants reported, and the larger they were in terms of actual body size (BMI). CDRS scores, however, did not significantly correlate with any of the *D*-IRAP scores, suggesting a lack of association between explicit and implicit measures of actual-ideal body size discrepancy. The only implicit score that correlated significantly with self-report measures was the Ideal-*D*_*fat*_ score (i.e. implicit desire to be/not be fat), with significant positive correlations with body image psychological inflexibility and symptoms of eating disorders, and a marginally significant (*p* = .062) positive correlation with body dissatisfaction as measured with the BSQ. Participants slower in affirming that they want to be fat and faster in denying it, were less flexible regarding their body image, more dissatisfied with it, and reported more symptoms of eating disorders.

In order to examine whether the implicit desire to (not) be fat could be used to make clinically relevant predictions regarding body image above and beyond explicit actual-ideal body image discrepancy (as measured with the CDRS), we conducted hierarchical regression analyses with body image psychological inflexibility (BI-AAQ scores), body dissatisfaction (BSQ scores), and eating disorder symptoms (EAT-40 scores) as the criterion variables. For each analysis, we compared a model in which the criterion variable was predicted by CDRS discrepancy scores with a model that also included Ideal-*D*_*fat*_ scores as a predictor. For all criterion variables, CDRS scores proved to be a statistically significant predictor, and the inclusion of Ideal-*D*_*fat*_ scores in the model produced a significant increment in predictive power (see Table 2). Overall, regression analyses showed that both the explicit discrepancy between current and desired body size as measured with the CDRS, and the implicit belief that I (don’t) want to be fat (as measured with the IRAP), independently contributed to predicting clinically relevant measures of body-image related distress.

**Table 2:** Hierarchical regression predicting body-image related psychological inflexibility (BI-AAQ), body dissatisfaction (BSQ), and eating disorder symptoms (EAT-40) from explicit actual-ideal body size discrepancy (CDRS discrepancy score) and implicit desire to (not) be fat (Ideal-*D*_*fat*_ score)

	B	SE	β	*t*	*F*	*R*^*2*^	∆R^2^
Dependent variable: BI-AAQ							
Step 1					21.009^***^	.167	–
CDRS	.223	.049	.408	4.584^***^			
Step 2					15.226^***^	.227	.060
CDRS	.231	.047	.422	4.883^***^			
Ideal*-D*_*fat*_	12.139	4.282	.245	2.835^**^			
Dependent variable: BSQ							
Step 1					46.141^***^	.305	–
CDRS	.611	.090	.553	6.793^***^			
Step 2					28.024^***^	.350	.045
CDRS	.624	.088	.564	7.127^***^			
Ideal*-D*_*fat*_	21.301	7.945	.212	2.681^**^			
Dependent variable: EAT-40							
Step 1					7.828^**^	.069	–
CDRS	.088	.031	.263	2.798^**^			
Step 2					7.001^**^	.119	.050
CDRS	.092	.031	.276	2.989^**^			
Ideal*-D*_*fat*_	6.701	2.779	.222	2.412^*^			

* p < .05; ** p < .01; *** p < .001

## Discussion

The pattern of overall *D*-IRAP effects in this study (both for the IRAP_ACTUAL_ and the IRAP_IDEAL_) is consistent with that observed in previous research [25], with small-sized positive general scores for both IRAP tasks (Actual-*D*_*general*_ and Ideal-*D*_*general*_), which are indicative of a slightly stronger identification with thinness and a slightly stronger desire to be thin (than to be fat). A more fine-grained analysis based on specific scores for thinness and fatness allows for a more nuanced interpretation of these overall findings. Consistent with our initial hypothesis, the results in the IRAP_ACTUAL_ show that participants were faster in identifying themselves (than not) with figural drawings of underweight. Likewise, as expected, participants did not reject self-identification with drawings of overweight/obesity. Furthermore, they did identify themselves with these drawings, but less than with drawings of underweight. This result is consistent with the fact that the sample was rather homogenous in terms of body mass index, with most participants within the range of normal weight. Since the drawings employed in the IRAP were representative of underweight and overweight/obesity, it is likely that for most participants none of the target drawings was representative of their actual body size. Yet, participants self-identified slightly more with the underweight than with the overweight drawings.. The results in the IRAP_IDEAL_ show the same pattern. Participants, as expected, were faster in affirming than denying that they want to be like the underweight drawings. Although they were also faster in affirming than denying that they want to be like the overweight drawings, the magnitude of this difference in response speed was smaller than for underweight drawings. Overall, the implicit desire to be thin was slightly stronger than the desire to be fat.

The lack of differential performance across both IRAP tasks suggests an absence of actual-ideal implicit discrepancy that to some extent contrasts with the observed actual-ideal explicit discrepancy measured with the CDRS, wherein participants indicated that they want to be thinner than they currently are. This is consistent with the lack of significant correlations between CDRS and IRAP scores in this study. It is important to note that prior IRAP research on body image also failed to find significant correlations between implicit and explicit measures [27, 30]. Typically, this inconsistency has been explained attending to the different patterns of relational responding brought about by each kind of task. According to the Relational Elaboration and Coherence model [40], implicit (e.g. reaction-time based tasks) and explicit (e.g. rating scales) procedures capture different patterns of relational responding. Although both types of procedures reflect the operation of the same behavioral process (i.e., arbitrarily applicable relational responding), depending on the properties of each assessment situation (e.g. time-pressured or not) two broad patterns of relational responding can be obtained. One, brief and immediate, reflects a strongly reinforced verbal response involving the stimuli presented in the task. The other, extended and elaborated, entails additional relational responding not only to the stimuli themselves, but also to the initial response to those stimuli. Without time constraints, these additional relational responses will form a relational network that needs to be coherent and consistent with other existing relational networks (which would include beliefs about what is socially valued/acceptable, or demand characteristics). In addition to time constraints, there is an important difference between the CDRS and the IRAP tasks in this study. Each trial in any of the IRAP tasks presented a single stimulus (isolated from the other drawings), so that the relational response required by the task was limited to affirming or denying a particular relation between “I” and the specific drawing presented (e.g. “I want to be” drawing X). However, the CDRS entailed responding to the nine stimuli ordered according to increasing body size. Thus, it involved responding to I-drawing relations, but always in the context of multiple comparative relations amongst the different drawings (e.g. “this one’s not bad, but the one on the left looks better”). The very conformation of the CDRS allowed for more elaborate responding, not only based on the lack of time-pressure demands, but because it involved a more complex relational target than the IRAP.

In any case, as mentioned above, the observed average explicit actual-ideal discrepancy as measured with the CDRS was small in magnitude (< 10% of the scale range) and indeed smaller than the distance between two adjacent figures in the scale. That is, participants explicitly expressed a desire to be just slightly thinner than they perceived themselves to be. Since the IRAP requires two clearly discriminable sets of target stimuli that can be contrasted (thus the drawings included in these IRAP tasks represent extremes of body size), it could be argued these IRAP tasks might not be particularly well suited to capture very small discrepancies around the normal weight range (like those observed with the CDRS in this study). Nonetheless, this was only the first approach to using the IRAP to assess actual-ideal body image discrepancy with figural drawings. Future studies might attempt to compare drawings that, though discriminable, are closer in the continuous.

In any case, it is worth mentioning that even with these constraints, the IRAP tasks in this study produced relevant outcomes with potential clinical implications. Both IRAP tasks addressed different, specific relations regarding the self and thinness/fatness. While the IRAP_ACTUAL_ focused on a relation of identification/non-identification with underweight or overweight/obesity, the IRAP_IDEAL_ aimed to capture relations of willingness/rejection to be underweight or overweight/obese. It was the latter, and specifically the implicit desire to (not) be fat (as measured with Ideal-*D*_*fat*_ score), that proved to be a key variable. While CDRS scores showed the strongest correlations with body-image distress self-report measures and BMI, Ideal-*D*_*fat*_ scores significantly correlated with body-image psychological inflexibility and eating disorder symptoms as well. Furthermore, regression analyses showed that both measures were independent predictors of body image self-report measures. The implicit desire to (not) be fat significantly added to the predictive power of a model that had the CDRS as predictor. This is interesting, since this specific verbal relation (wanting/not wanting to be fat) seems to reflect key aspects of body image psychological flexibility, like willingness to have a body that does not conform to the thin ideal. While IRAP research has repeatedly shown an absence of negative bias towards fatness at the group level [26, 27, 30, 31], it is individual variation in brief, immediate verbal responses regarding fatness that seems to play a significant role in the prediction of different clinically relevant body-image outcomes, as shown in the present study and in previous work [27]. In any case, it would be interesting to further examine this idea in future research on specific implicit beliefs about fatness with clinical populations.

Although this study was not specifically designed to examine gender differences, the exploratory comparisons we conducted revealed some interesting results. The difference between females and males in actual-ideal discrepancy measured with the CDRS did not reach significance (contrary to what we hypothesized). However, men showed a significantly stronger implicit desire to be fat than women. That is, in time-pressure conditions, male participants were more open (or less reluctant) to the idea of becoming fatter. This latter result is consistent with the evidence pointing out that females are typically under stronger social pressure than males to lose weight and attain a slim figure, with females showing stronger idealization of the thin ideal [41]. Although this finding underscores the potential of implicit measures for the study of gender differences regarding clinically relevant body image beliefs, further research is needed.

This study presents some limitations that we would like to comment. First, although research using the IRAP for the study of body image is growing, it has to be admitted that it is still a relatively new instrument. While it has been pointed out as one implicit measure with strong predictive validity [42, 43], it has also been noted that more information is needed on some psychometric properties like test-retest reliability [44]. Future studies on body image might consider administering the IRAP tasks on repeated occasions in order to quantify this form of reliability. Second, our sample’s data for height and weight (upon which BMI was calculated), as well as the presence/absence of a diagnosis of eating disorders were based on self-report, which is potentially subject to self-presentation bias. No participants in our sample reported an eating disorder diagnosis, which is relevant considering a recent estimation of 2.2% point prevalence for accurately diagnosed eating disorders in Europe [45]. Future studies might collect actual weight and height, and introduce a formal assessment for eating disorders as a criterion for sample selection, instead of relying exclusively on self-report for these data.

## Conclusions

The present study set out to examine the discrepancy between actual and ideal body image with the IRAP using target stimuli like those in traditional explicit measures of body-image discrepancy, analyzing beliefs specifically related to thinness or fatness. While the IRAP results were not indicative of an implicit actual-ideal discrepancy, specific implicit beliefs about fatness (wanting/not wanting to be fat) emerged as a relevant variable in predicting body dissatisfaction and eating disorder symptoms, and particularly body-image psychological inflexibility. In our view, these findings suggest that prevention efforts for eating disorders should focus on promoting willingness to accept the idea that one can have a larger body size, and a more flexible sense of self that is not entirely built upon body image, but includes multiple other aspects and roles that, like body image itself, may change throughout the course of life.

## Data Availability

The datasets used and/or analysed during the current study are available from the corresponding author on reasonable request.

## Abbreviations

IRAP: Implicit Relational Assessment Procedure
IRAP_ACTUAL_: First IRAP task, assessing actual body image, with labels I am / I am not.
IRAP_IDEAL_: Second IRAP task, assessing ideal body image, with labels I want to be / I don’t want to be
CDRS: Contour Drawing Rating Scale
RFT: Relational Frame Theory
BMI: Body mass index
BI-AAQ: Body image-Acceptance and Action Questionnaire
BSQ: Body Shape Questionnaire
EAT: Eating Attitude Test
Actual-*D*_*thin*_: Specific *D*-IRAP score for the IRAP_ACTUAL_ based only on trials including underweight drawings
Actual-*D*_*fat*_: Specific *D*-IRAP score for the IRAP_ACTUAL_ based only on trials including overweight/obese drawings
Actual-*D*_*general*_: Overall *D*-IRAP score for the IRAP_ACTUAL_ based on all trials
Ideal-*D*_*thin*_: Specific *D*-IRAP score for the IRAP_IDEAL_ based only on trials including underweight drawings
Ideal- *D*_*fat*_: Specific *D*-IRAP score for the IRAP_IDEAL_ based only on trials including overweight/obese drawings
Ideal-*D*_*general*_: Overall *D*-IRAP score for the IRAP_IDEAL_ based on all trials
